# Improvement of the Heat-Dissipating Performance of Powder Coating with Graphene

**DOI:** 10.3390/polym12061321

**Published:** 2020-06-10

**Authors:** Fei Kung, Ming-Chien Yang

**Affiliations:** Department of Materials Science and Engineering, National Taiwan University of Science and Technology, Taipei 10607, Taiwan; philip@allightec.com

**Keywords:** graphene, powder coating, thermal conductivity, heat dissipation, thermal radiation

## Abstract

In this study, the epoxy powder was blended with graphene to improve its thermal conductivity and heat dissipation efficiency. The thermal conductivity of the graphene-loaded coating was increased by 167 folds. In addition, the emissivity of the graphene-loaded coating was 0.88. The epoxy powder was further coated on aluminum plate through powder coating process in order to study the effect on the performance of heat dissipation. In the case of natural convective heat transfer, the surface temperature of the graphene-loaded coated aluminum plate was 96.7 °C, which was 27.4 °C lower than that of bare aluminum plate (124.1 °C) at a heat flux of 16 W. In the case of forced convective heat transfer, the surface temperature decreased from 77.8 and 68.3 °C for a heat flux of 16 W. The decrease in temperature can be attributed to the thermal radiation. These results show that the addition of graphene nanoparticles in the coating can increase the emissivity of the aluminum plate and thus improving the heat dissipation.

## 1. Introduction

Heat-dissipating coating is important for the stabilization and miniaturization of electronic components. As the aggregate density and power intensity of electronic components continue to increase, large amount of heat generated from these devices must be dissipated in a timely manner. However, the heat dissipation performance of today’s electronic components cannot meet the requirements, thereby limiting the efficiency and service life of certain electronic components. To resolve this problem, heat-dissipating coating enhances the heat dissipation efficiency of the surface of a component [1]. It lowers the temperature of the heat-generating component in time and hence extends the service time and stability of components.

Literatures and patents on graphene heat-dissipating powder coating have been sparse; most of them confuse “heat dissipation” with “heat conduction” [2]. In general, the most important functions of a heat dissipation module in an electronic product include not only a rapid transfer of heat from the thermal source to the surface of the heat sink but also the ability to quickly disperse heat into the atmosphere through convection and radiation. A high thermal conductivity can only solve the problem of quick heat conduction. On the other hand, heat dissipation depends mainly on the heat dissipation area, profile, natural convection, and thermal radiation of the heat sink; it almost has nothing to do with the thermal conductivity of materials. Therefore, as long as the thermal conductivity is adequate, heat-dissipating coating can still be used as good heat dissipation modules for electronic products. Proper structural design of product or module can easily achieve a large heat dissipation surface area for convection. However, to achieve high heat dissipation efficiency through radiation, high thermal radiation coefficient is necessary [3].

Graphene is a nanomaterial with only one layer of carbon atoms. It features low density, low chemical activity, high thermal conductivity, large specific surface area, and high infrared emissivity. Graphene has superior heat conduction characteristics and its thermal radiation coefficient is greater than 0.95 [4]. Balandin et al. reported the thermal conductivity of suspended single-layer graphene measured near 5000 W m^−1^ K^−1^, which is one of the highest thermal conductivity of the currently known materials [5]. Therefore, from the perspective of heat conduction, heat dissipation, or thermal management, graphene can effectively improve the heat dissipation performance of existing thermal dissipation products for electronic components, assemblies, and LEDs as long as graphene products can be configured to meet the design requirements. However, the stacking tendency of graphene led to poor dispersion and greater post-processing difficulties, thereby preventing graphene from exhibiting its superior characteristics [6,7].

Thermoset powder coating comprises thermoset resin, hardener, dye, filler, and additives. There are several types of thermoset powder coatings: epoxy resin, polyester, and acrylic resin. Table 1 compares the pros and cons of these three types of powder coatings. The constituents are first mixed according to a specific ratio, followed by hot extrusion and crushing and other preparation processes. The coating is then applied by an electrostatic spray or friction spray (a thermoset method) at ambient temperature. It is then baked, melted, and cured to form a shiny permanent coating for heat dissipation and corrosion prevention. [8,9]. Powder coating generally has a better thermal conductivity than solvent coating due to the better binding between the coating and the substrate. More thoroughly cured coating leads to more stable crosslinking and hence denser and tighter coating [10,11]. This favors the reduction of scattering in the “lattice vibration” of the thermal dissipation mechanism.

The discussion of radiation and convection is rare. This study is aiming to investigate the enhancing effect of graphene-loading on the thermal dissipation performance of aluminum plate. The aluminum plate was attached to a heater as the heat source. The heat was transferred through the Al plate to the ambient atmosphere via convection and radiation. The plate was either bare or coated with a thin layer of polymer filled with graphene nanoflakes or boron nitride nanoparticles. The performance of the heat dissipation was evaluated by measuring the surface temperature on the plates with or without coating at a constant heat flux under forced convection or natural convection conditions. This study will demonstrate the significance of radiation heat transfer in the heat dissipation.

## 2. Materials and Methods

### 2.1. Materials

Graphene (AG05, grain size 5 μm, thickness 3.5 nm, aspect ratio 1429) was supplied by Allightec Co., Taichung, Taiwan. Aluminum plates (AL101001, Kuopont Chemical, Taoyuan, Taiwan) were used as the substrate for coating. The dimensions of the plate were 10 × 10 × 0.1 cm^3^. Epoxy resin (E12(604), Dow Chemical, Midland, MI, USA) and polyester (SJ4ET, Shenjian New Materials, Wuhu, China) were used as the matrix of the coating. Furthermore, hardener (HR0001, Kuopont Chemical, Taiwan) and additive (AD0001 Chemical, Kuopont, Taoyuan, Taiwan) were employed to give the coating (Table 2) both chemical resistance and weather resistance.

### 2.2. Preparation of Powder Coating

All the ingredients were blended using a single-screw extruder (PK–55, Pinying Machine Co., Kaohsiung, Taiwan) at 85–90 °C and a screw speed of 60 rpm. The resultant blend was pressed into sheets using roller miller and ground into powder (diameter: 0.1–2 μm) using a milling machine (SFM–22, Shehui Co., Taoyuan, Taiwan). The powder was deposited directly onto the substrate surface through electrostatic spraying using a sprayer (PEM–X1, Wagner, Markdorf, Germany) before curing at 160–200 °C.

### 2.3. Measurement of Thermal Conductivity

The thermal conductivity was determined using a thermal conductivity meter (LFA447 NanoFlash, Netzsch, Selb, Germany). Thermocouples were attached to the surface of the specimens. The coating contained 3 wt% of either multilayer graphene, boron nitride, or without additive as the control. The thermal conductivity of the coating was calculated according to the following equation:

(1) $$\frac{L_{T}}{k_{T}}=\frac{L_{1}}{k_{1}}+\frac{L_{2}}{k_{2}}$$

where *L*_1_, *L*_2_ and *L*_T_ are the thicknesses of the coating, the substrate and the total thickness, respectively, and *k*_1_, *k*_2_ and *k*_T_ are the thermal conductivities of the coating, the substrate, and the overall thermal conductivity, respectively. The thickness of the aluminum plate was 1 mm, whereas that of the coating was measured using a coating thickness meter (Qnix Qua Nix 4200P, Automation Dr. Nix GmbH & Co. KG, Cologne, Germany). The coating thickness was 40 μm.

### 2.4. Measurement of Thermal Emissivity

The thermal emissivity was measured using an infrared emissivity detector (ED01, Conjutek Co., New Taipei City, Taiwan) in the wavelength range of 2 to 22 μm.

### 2.5. Forced Convective Heat Transfer

**Figure 1:**
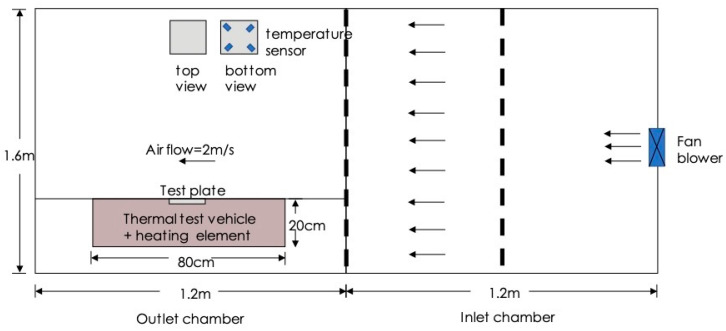
The experimental setup for conducting forced convection.

The forced convective heat transfer of the coated and bare plates was performed according to the standard of AMCA 210–07. Figure 1 depicts the experimental setup for conducting forced convection. The heat supply was set either 8 W or 16 W. The plate was placed horizontally under a flow rate of 2 m/s. Temperatures were measured at four points on the bottom surface of the plate.

### 2.6. Natural Convective Heat Transfer

**Figure 2:**
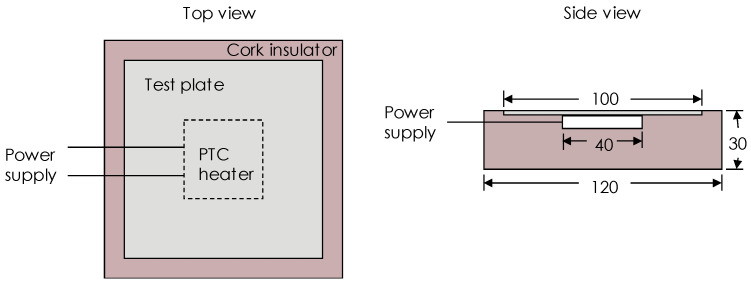
The experimental setup for conducting natural convection.

The natural convective heat transfer was performed by placing the plate horizontally as illustrated in Figure 2. The temperature was monitored until reaching steady state.

## 3. Results and Discussion

### 3.1. Characteristics of Graphene and Powder Coating

**Figure 3:**
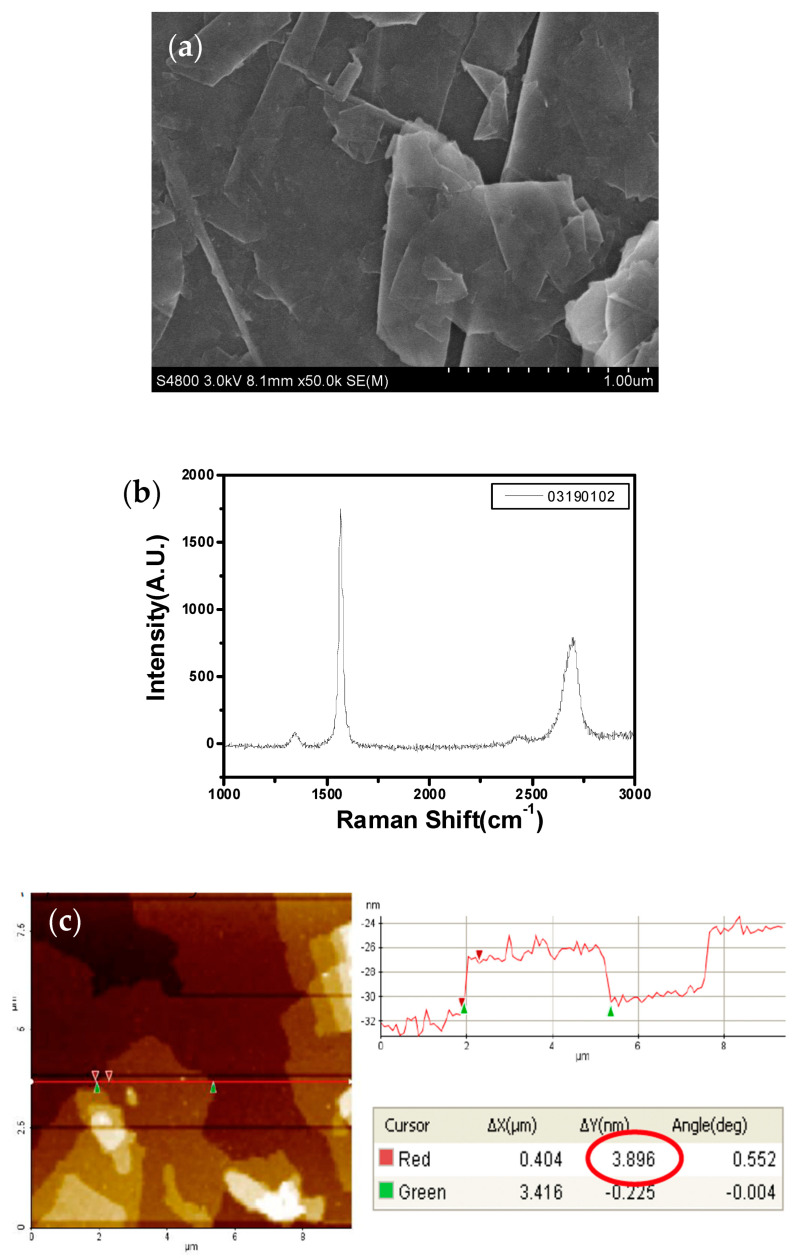
The characteristics of graphene nanoparticles. (**a**) SEM image; (**b**) Raman spectrum; (**c**) AFM image.

Table 3 and Figure 3 show the characteristics of the graphene obtained from the supplier. From the Raman spectrum of graphene, there are three distinct absorption peaks: D peak at 1353 cm^−1^, G peak at 1581 cm^−1^, and 2D peak at 2720 cm^−1^. The I_D_/I_G_ is about 0.05 and the I_2D_/I_G_ is about 0.36, indicating that this is multilayer graphene. The AFM image shows that the horizontal dimension of the graphene sheet is between 3–25 μm.

**Figure 4:**
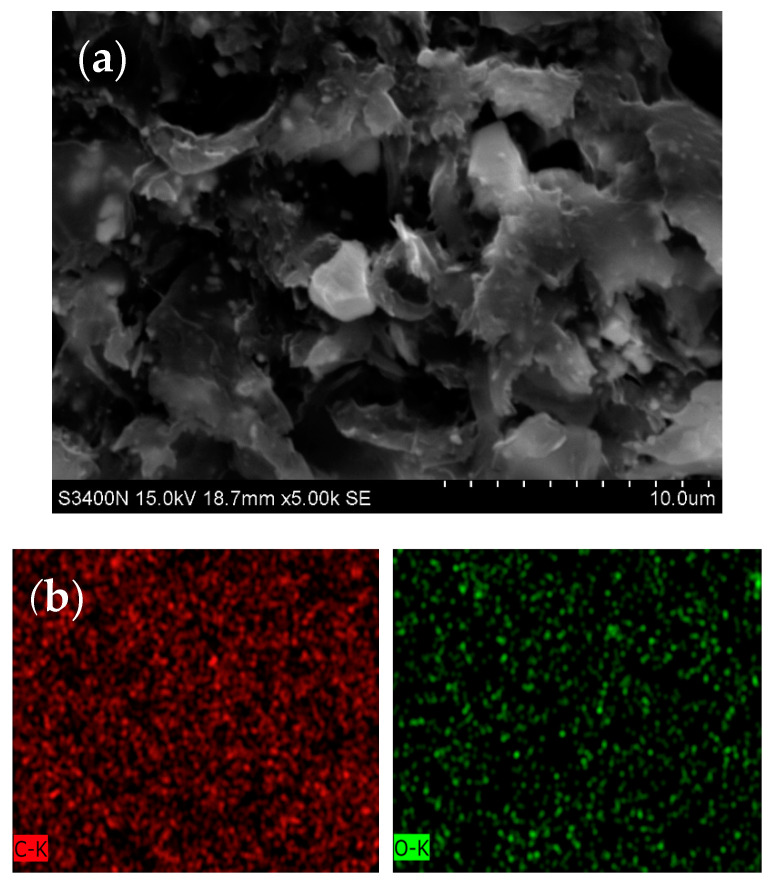
The SEM image of the graphene-loaded coating. (**a**) The micrograph of cross-section; (**b**) EDS images of carbon and oxygen.

Figure 4 shows the SEM image of the cross section of graphene-loaded coating as well as the EDS images of carbon and oxygen. These images indicated that graphene nanoparticles were well distributed in the coating matrix. Furthermore, Table 4 shows that the carbon content in the coating with graphene was slightly higher than that in the pristine coating, indicating the presence of graphene. Some micro-scale aggregates were observable in Figure 4a. Similar observation was also reported in the literature [12]. This may affect the thermal conductivity of the coating, however, it is out of scope of this study.

Graphene loaded nanocomposites have been considered for thermal managements. There are several reviews regarding the thermal conductivity of graphene-polymer composites [13,14,15]. In recent years, graphene and expanded graphite have been widely studied as nanofillers for polymer composites, as thermal interface materials and heat sinks [16,17,18,19]. In addition to the extremely high thermal conductivity of single-layer graphene, two-dimensional morphology also makes graphene more conducive, thus improving heat transfer performance. The thermal conductivity of graphene-polymer composites is affected by factors including loading, graphene orientation, and interface [20]. Graphene exhibits a very high specific surface area leading to large interface with the polymer chains, and causing phonon scattering and hence ultra-high interface thermal resistance. Therefore, heat is difficult to transfer through the graphene-polymer interface. In addition, when the loading of graphene is above the percolation threshold, the thermal conductivity of this composite would be increased significantly. When the orientation of graphene is in the direction of heat flow, facilitating the formation of thermal conductive channel and hence improve the thermal conductivity. However, in this study, the powder was deposited onto the substrate through electrostatic spraying, thus these graphene nanosheets were randomly oriented.

This study chose thermoset powder coating as the research object. A thermoset resin is used as the film forming material, and a hardener with a crosslinking reaction is added to form an insoluble, non-melting hard coating after heating. Such a coating would not soften like thermoplastic coating even at elevated temperatures; it can only fracture. Since the resin used in the thermoset powder coating is a low molecular weight pre-polymer with a low degree of polymerization, it has good leveling and decorative properties. Moreover, this low molecular weight pre-polymer can be crosslinked into 3D network after curing, endowing the coating good corrosion resistance and mechanical properties. This has led to rapid development of the thermoset powder coating.

### 3.2. Thermal Conductivity

Table 5 shows the thermal conductivities of the coated and uncoated aluminum plates. The overall thermal conductivity was reduced from 196.7 W/m-K of the bare aluminum to 88.2 W/m-K of the epoxy/BN coated aluminum plate. This indicates that the coating on the surface can impair the heat conduction. This may appear to violate the purpose of improving thermal dissipation. However, the heat generated from the electronic elements dissipates to the ambient through not only conduction but also convection and radiation. In the subsequent sections, the coating actually did facilitate the dissipation of the heat.

The thermal conductivity of the coating in Table 5 was calculated from the overall thermal conductivity according to Equation (1). Three types of coating were measured: pristine epoxy-polyester coating (EPC), BN-loaded (EBN) and graphene-loaded (EGR) epoxy-polyester coating. The thermal conductivity of the BN-loaded coating was slightly higher than that of the pristine epoxy coating. On the other hand, the loading of graphene improved the thermal conductivity of the coating to above 6 folds. This is reasonable since graphene is well-known for high thermal conductivity. Because the pristine epoxy-polyester coating exhibited low thermal conductivity, this coating was not studied further in the subsequent heat transfer experiments. Only Al, EBN and EGR were employed in the heat transfer tests.

### 3.3. Thermal Emissivity

Table 6 shows the emissivity of the samples in the wavelength range from 2 to 22 μm. In general, the emissivity values of metals are low while those of polymers are much higher. In this study, EBN coating appears white, whereas EGR coating appears black.

### 3.4. Forced Convective Heat Transfer

In order to investigate the role of radiation heat transfer in the thermal dissipation performance of coating, the aluminum plates were subject to heat transfer experiments under natural convection and forced convection.

Table 7 summarizes the results of heat transfer under forced convection. For a small object in a big room, the radiative heat flux was calculated according to the Stefan-Boltzmann Law: [21]

(2) $$q_{r}=\varepsilon \sigma (T_{s}^{4}-T_{a}^{4})$$

where *q_r_* is the radiative heat flux from the sample to the ambient, *ε* is the emissivity of the surface, σ is the Stefan-Boltzmann constant (5.67 × 10^8^ W/m/K^4^), and *T_s_* and *T_a_* are respectively the surface temperature and ambient temperature (in K). The convective heat flux (*q_c_*) equals the total heat flux (*q_t_*) minuses the radiative heat flux. The radiative heat transfer ratio is *q_r_*/*q_t_*. For bare aluminum plate, because of low emissivity, the radiative heat transfer ratio was 1.7–1.9%. However, for aluminum plates coated with epoxy-polyester resin loaded with BN or graphene, the radiative heat transfer ratio increased to 8.9–9.4% and 15.9–16.6%, respectively. These additional heat flux would improve the heat dissipation, making the surface temperature lower, thus the heating source (e.g., IC or LED) would be cooler. Indeed, the surface temperature for EGR were 7 °C and 13 °C lower than those for bare aluminum when the heat flux was respectively 800 and 1600 W/m^2^.

**Figure 5:**
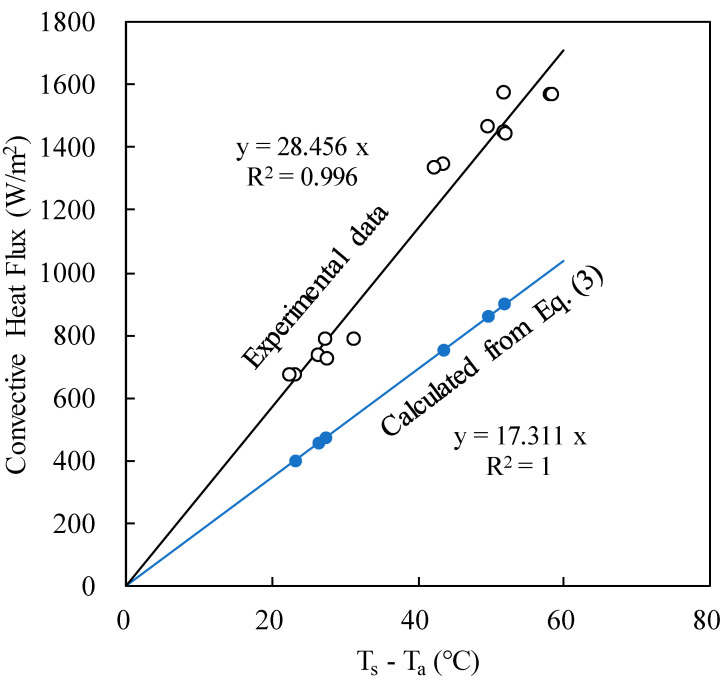
The linear correlation between convective heat flux and temperature difference.

Figure 5 shows that the convective heat flux depends linearly with the temperature difference. The slope (28.456 W/m^2^K) is the convective heat transfer coefficient under this specific test condition. The coefficient of determination (R^2^) was 0.996, indicating that this correlation fits very well to the experimental results. We can use this value to predict the heat dissipation rate at other heat flux at the same air flow speed. Furthermore, the heat transfer coefficient is independent on the substrate, whether it is bare aluminum or coated with a layer of polymer coating.

The Reynolds number Re (= *uL*/*ν*) for this test condition was around 1.2 × 10^4^, less than 5 × 10^5^, suggesting the air flow was laminar. For laminar forced convection, the heat transfer coefficient based on boundary layer model is as follows

(3) $$h_{L}=0.664{Pr}^{1/3}{Re}_{L}^{1/2}\left(\frac{k}{L}\right)$$

where *L* is the length of the plate, *k* is the thermal conductivity of air, *Pr* is the Prandtl number of the air, *Re* is the Reynolds number of the air stream, *u* is the speed of the air stream, and *ν* is the kinematic viscosity the air. The resultant convective heat flux was then calculated as

(4) $$q_{fc}=h_{L}\left(T_{s}-T_{a}\right)$$

The calculated results were presented in Figure 5 as well. However, the heat transfer coefficient (the slope) was only 60% of the experimental results. This probably is due to the turbulence in the actual measuring environment, which would accelerate heat transfer.

### 3.5. Natural Convective Heat Transfer

In addition to forced convection, natural convection is the other path for heat dissipation. Table 8 summarizes the results of heat transfer under natural convection. All the conditions were the same as in Section 3.4, except there was no air flowing on the surface. The temperature difference was higher that its counterpart in Table 7, suggesting that natural convection is slower than forced convection in heat dissipation. Furthermore, because of higher surface temperature, the radiative heat flux in natural convection was higher than in forced convection. Consequently, the convective heat flux in natural convection was lower than in forced convection, reflecting the slower heat dissipation in natural convection. The order of the radiative heat transfer ratio was the same as in Table 7, that is, EGR > EBN > Al. This order is the same as that of the emissivity, suggesting that graphene-loaded coating can enhance heat dissipation.

**Figure 6:**
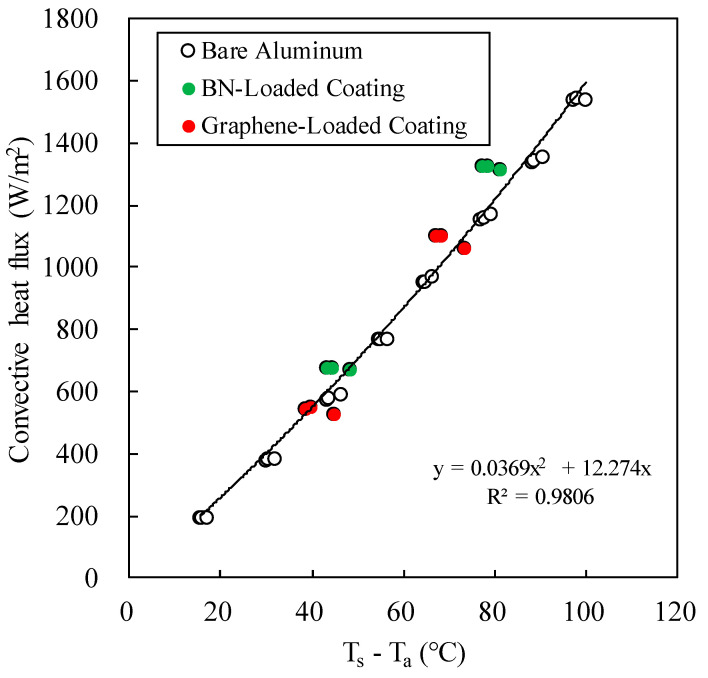
The convective heat fluxes of coated and uncoated aluminum plates under natural convection.

Natural convection is a result of the motion of the fluid due to density changes arising from the heating. In this study, the heated plate was placed horizontally, inducing an upward air stream. The flow pattern is complicate. No reliable empirical correlation is capable to predict the heat transfer. Therefore, we construct an empirical correlation of convective heat flux vs temperature difference. Because the aluminum plate has a low emissivity, the aluminum plate was used to measure the surface temperature for a series of total heat fluxes. The convective heat flux was obtained by subtracting the radiative heat flux from the total heating flux. Figure 6 shows that the convective heat flux depends on the temperature difference. Linear regression yielded a quadratic correlation with R^2^ equals to 0.981.

*q*_c_ = 0.0369(Δ*T*)^2^ + 12.27Δ*T* (5)

### 3.6. Heat Transfer Coefficients

Table 9 summarizes heat transfer coefficients calculated from the experimental results in Table 7 and Table 8. Heat transfer coefficient is the measure of heat dissipation. Among these heat transfer coefficients, the total heat transfer coefficient (*h*_T_) was calculated as follows:

*h*_T_ = *q*_T_/Δ*T* (6)

and the convective heat transfer coefficient (*h*_c_) and the radiative heat transfer coefficient (*h*_r_) were calculated respectively as follows:

*h*_c_ = *q*_c_/Δ*T* (7)

*h*_r_ = *q*_r_/Δ*T* (8)

where Δ*T* is the temperature difference between the surface temperature and the ambient temperature.

These three heat transfer coefficients are affected by three factors: type of convection, surface coating, and total heat flux. The weight of each factor on each coefficient can be evaluated statistically with analysis of variance (ANOVA).

#### 3.6.1. Total Heat Transfer Coefficient

Table 10 presents the results of ANOVA for total heat transfer coefficient. The results show that all three factors significantly affect *h*_T_. Among these factors, the type of convection was the most influential while *q*_T_ was the least.

**Figure 7:**
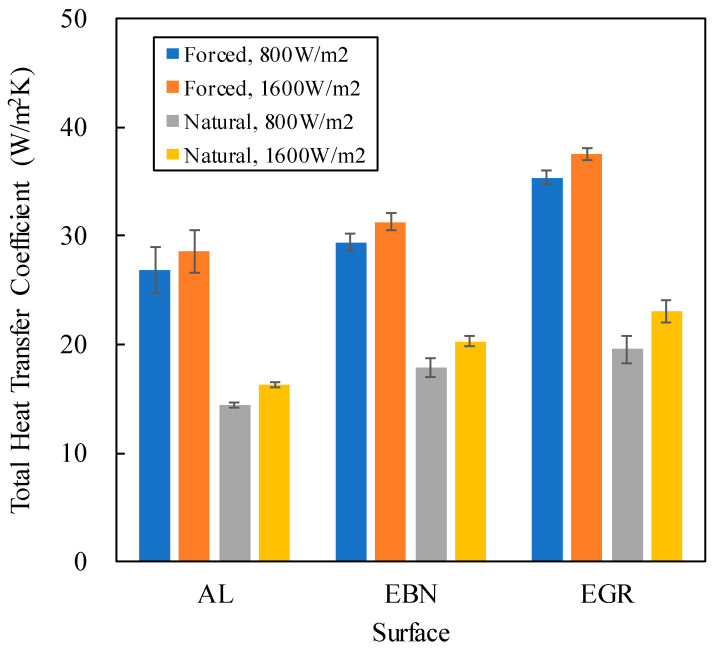
Effect of surface type on the total heat transfer coefficient.

Figure 7 shows that the total heat transfer coefficient of the forced convection was about twice of that of the natural convection. This reflects the fact that forced convection can remove heat faster than natural convection. Furthermore, bare aluminum surface exhibited lower ***h***_T_ and ***h***r than the other two coated surfaces. This can be attributed to the faster radiative heat transfer from coated aluminum plates, and that graphene-loaded coating exhibited higher ***h***_T_ than other surfaces, since the emissivity of EGR was much higher than others. Figure 7 also shows that higher total heat flux (***q***_T_) led to higher *h*_T_ for each surface. In forced convection, the increase was at most 6%, whereas in natural convection, the increase jumped to 17%. However, the effect of ***q***_T_ was less than the effect of the surface, which is consistent with ANOVA.

#### 3.6.2. Convective Heat Transfer Coefficient

**Figure 8:**
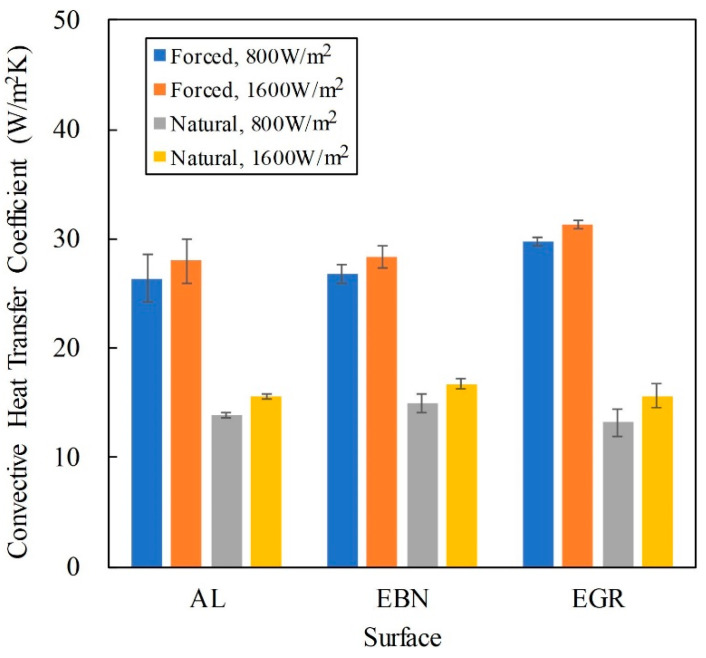
Effect of surface type on the convective heat transfer coefficient.

Table 11 shows that the major factor affecting *h*_c_ was the type of convection_._ Figure 8 also shows that the *h*_c_ of forced convection was about twice of that of natural convection This is expected because *h*_c_ is the “convective” heat transfer coefficient. The type of surface coating affects less significantly to *h*_c_. This is obvious because thermal radiation depends only on the temperature difference and would not affect the air flow.

The ANOVA results indicated that *q*_T_ was the minor factor for *h*_c_. This is supported in Figure 8 that higher q_T_ led to slightly higher *h*_c_. In forced convection, according to Equation (3), the convective heat transfer coefficient is proportional to the thermal conductivity of the air, which increases with the temperature. Because the surface temperature increased with the total heat flux, leading to higher thermal conductivity and hence higher *h*_c_. However, the increase in *h*_c_ was small, thus the slope of q_c_ in Figure 5 was a constant, suggesting a constant *h*_c_.

In natural convection, Figure 6 shows that *q*_c_ is a quadratic function of Δ*T*, thus *h*_c_ is a linear function of Δ*T*:

*h*_c_ = 0.0369(Δ*T*) + 12.27 (9)

However, the prefactor 0.0369 was small, making a weak dependency of *h*_c_ on ΔT.

#### 3.6.3. Radiative Heat Transfer Coefficient

**Figure 9:**
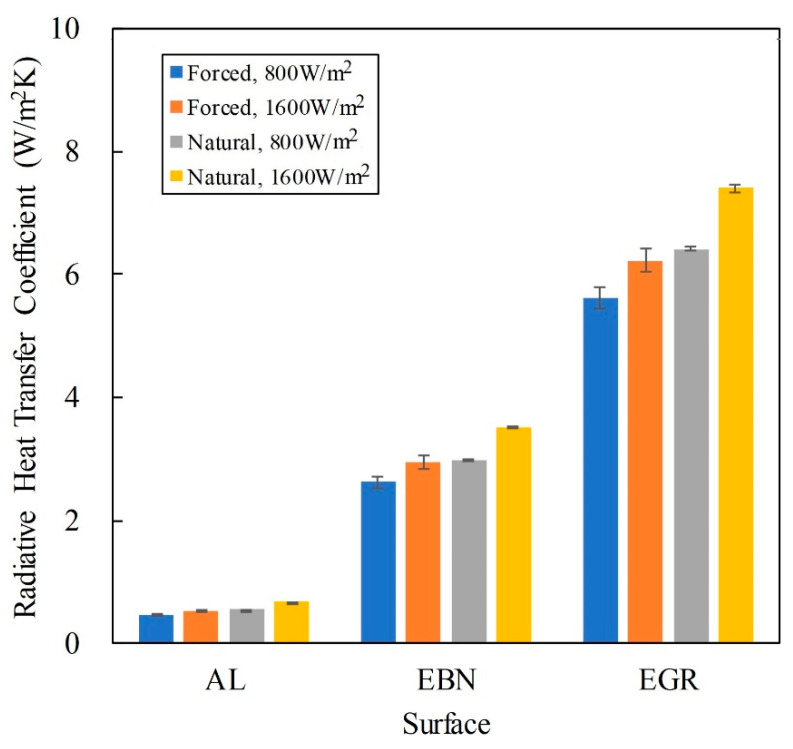
Effect of surface type on the radiative heat transfer coefficient.

Table 12 shows the ANOVA results and that for *h*_r,_ the major factor is the surface coating and the minor factor is the type of convection. The total heat flux affected the least the radiative heat transfer. The radiative heat transfer increased with the emissivity of the surface. In this study, the emissivity varied greatly, ranging from 0.07 for aluminum, 0.4 for BN-loaded coating, to 0.88 for graphene-loaded coating. Thus, the effect of emissivity on *h*_r_ is significant. Figure 9 also shows this effect. The type of convection affected *h*_r_ through *T*_s_ and *T*_a_, because *h*_r_ can be calculated as follows:

*h*_r_ = *σ*ε (*T*_s_^2^ + *T*_a_^2^) (*T*_s_ + *T*_a_) (10)

The surface temperature was lower for forced convection because of higher *h*_c_.

Figure 9 summarizes the effect of surface coating on *h*_r_. The difference between convection types was less than that between surfaces. The effect of *q*_T_ was further lower than the effect of convection.

## 4. Conclusions

The transfer of heat from the source (IC, LED, etc.) to the sink (ambient) involves both heat convection and heat conduction. There is another route for heat dissipation occurring in the ambient, that is, radiation, as long as the surface temperature is different to the ambient temperature. In nature and in engineering, the natural cooling or heating of objects is achieved by natural convection heat transfer. The intensity of natural convection heat transfer is weak, especially in the air environment, with radiation heat transfer of the same order of magnitude. At relatively high temperatures, the intensity of radiative heat transfer is much stronger than that of natural convective heat transfer. Therefore, in the actual calculation of natural convective heat transfer, radiative heat transfer should not be neglected.

In this study, graphene nanoparticles were blended into epoxy-polyester powder. Aluminum plate was then coated with aforementioned powder blends. For comparison, BN-loaded coating plates were also prepared. The thermal conductivity of the coating was improved from 5 W/m∙K to 6 and 33.3 W/m-K for the BN- and graphene-loaded coating, respectively. The performance of heat dissipation of the resulting plates was further investigated under forced and natural convection. Under the forced convection, the radiative heat transfer coefficient (*h*_r_) of the bare Al plate took about 1.8% of the total heat transfer coefficient (*h*_T_), whereas for the graphene-loaded coating, *h*_r_ took about 16% of *h*_T_. Therefore, radiative heat transfer is not negligible in heat dissipation through forced convection.

Under the natural convection, the *h*_r_ of bare Al plate was about 4% of *h*_T_, while the *h*_r_/*h*_T_ of graphene-loaded coating was about 33%, indicating that the thermal radiation cannot be ignored in the dissipation through natural convection.

The heat dissipation in this study showed that thermal radiation is a non-negligible route under either forced convection or natural convection. Based on this finding, a thin layer of graphene-loaded coating with a high emissivity can improve the heat dissipation performance of metal substrate.

## Figures and Tables

**Table 1:** Types and surface characteristics of resins.

	Epoxy	Epoxy Polyester	Polyester
Hardness	Excellent	Very good	Very good
Softness	Excellent	Excellent	Excellent
Baking resistance	Very Poor	Very good	Excellent
Weatherability	Poor	Poor	Excellent
Corrosion resistance	Excellent	Very good	Very good
Chemical resistance	Excellent	Good	Very good
Operability	Very good	Excellent	Excellent

**Table 2:** Composition of powder coatings.

Ingredient		Product ID	Content, wt%	Manufacturer
Epoxy resin		E12–604	33	Shang-shan, Dow Chemical, Midland, MI, USA
Polyester resin		SJ4ET	35	Shen-Jian, Wuhu, China
Curing agent		KPC–03	6	Kuopont, Taoyuan, Taiwan
Auxiliary		KPA–01	5	Kuopont, Taoyuan, Taiwan
TiO_2_		BLR-698	18	Lomon Billions, Jiaozuo, China
Filler	Graphene	AG05	3	Allightec, Taichung, Taiwan
	Boron nitride	TSD–03	3	Topspin, Kaohsiung, Taiwan

**Table 3:** Characteristics of graphene nanoparticles.

Item	Properties	Test Method
appearance	Black Granules	visual
lateral size (μm)	3–25	particle analyzer
number of layers	6–10	AFM
carbon content (%)	>99.5	X-ray photo-electronic spectroscopy
oxygen content (%)	<0.1	X-ray photo-electronic spectroscopy
water adsorption content (%)	≤0.5	ASTM D570–2005
bulk density (g/cm^3^)	0.03–0.1	powder densitometer
true density (g/cm^3^)	2.25	density tester
specific surface area (m^2^/g)	25–50	specific surface area tester

**Table 4:** Atomic compositions of the coatings with or without graphene from EDS results.

Element	Pristine Coating	Graphene-Loaded Coating
C (mol%)	75.7	77.4
O (mol%)	24.3	22.6

**Table 5:** Thermal conductivity of the aluminum plates with or without coating *.

	Sample	Al (Bare Aluminum Plate)	EPC (Epoxy-Polyester Coating)	EBN (Boron Nitride-Loaded Coating)	EGR (Graphene-Loaded Coating)
K (W/m-K)					
Overall		196.7	79.5	88.2	165.0
Coating		-	5.0	6.0	33.3

* *T* = 25 °C, Light voltage = 250 V, pulse width = 0.02 ms, model = Cowan.

**Table 6:** The emissivities of aluminum plate and two types of coatings *.

Test Item	Al	EBN	EGR
Emissivity, ε	0.07	0.40	0.88

* *T* = 25 °C, test time = 3 s.

**Table 7:** The heat transfer rates by convection and radiation under forced convection *.

Surface	Al	Al	EBN	EBN	EGR	EGR
Total heat flux, q_T_ (W/m^2^)	800	1600	800	1600	800	1600
Temperature difference, ΔT (°C)	29.9 ± 2.3	56.3 ± 3.8	27.2 ± 0.8	51.2 ± 1.3	22.6 ± 0.4	42.7 ± 0.7
Radiative heat flux, q_r_ (W/m^2^)	14 ± 2	30 ± 3	71 ± 4	151 ± 10	127 ± 2	266 ± 4
Convective heat flux, q_c_ (W/m^2^)	786 ± 2	1570 ± 3	728 ± 4	1450 ± 11	673 ± 2	1336 ± 6
Radiative heat transfer ratio, %	1.7 ± 0.2	1.9 ± 0.2	8.9 ± 0.6	9.4 ± 0.6	15.9 ± 0.2	16.6 ± 0.2

* RH = 76.2%, *P*_amb_ = 747.5 mm Hg, air flow rate = 2 m/s.

**Table 8:** The heat transfer rates by convection and radiation under natural convection.

Surface	Al	Al	EBN	EBN	EGR	EGR
Total heat flux, q_T_ (W/m^2^)	800	1600	800	1600	800	1600
Temperature difference, ΔT (°C)	55.4 ± 1.0	98.5 ± 1.2	45.2 ± 2.6	79.0 ± 2.1	41.0 ± 3.2	69.6 ± 3.2
Radiative heat flux, q_r_ (W/m^2^)	31 ± 1	67 ± 1	135 ± 8	278 ± 8	263 ± 22	515 ± 29
Convective heat flux, q_c_ (W/m^2^)	767 ± 2	1538 ± 2	673 ± 1	1321 ± 5	538 ± 13	1086 ± 25
Radiative heat transfer ratio, %	3.8 ± 0.1	4.1 ± 0.1	16.7 ± 0.9	17.4 ± 0.5	32.8 ± 2.3	32.2 ± 1.7

**Table 9:** Comparison of heat transfer coefficients in forced and natural convection.

	Forced Convection						Natural Convection					
Surface	AL	EBN	EGR	AL	EBN	EGR	AL	EBN	EGR	AL	EBN	EGR
q_T_ (W/m^2^)	800	800	800	1600	1600	1600	800	800	800	1600	1600	1600
*h*_T_ (W/m^2^K)	26.8 ± 2.1	29.4 ± 0.8	35.4 ± 0.6	28.5 ± 2.0	31.3 ± 0.8	37.5 ± 0.6	14.4 ± 0.2	17.9 ± 0.9	19.6 ± 1.3	16.3 ± 0.2	20.2 ± 0.5	23.0 ± 1.0
h_c_ (W/m^2^K)	26.4 ± 2.1	26.8 ± 0.9	29.7 ± 0.4	28.0 ± 2.0	28.3 ± 0.9	31.3 ± 0.4	13.8 ± 0.2	14.9 ± 0.9	13.2 ± 1.3	15.6 ± 0.2	16.7 ± 0.5	15.6 ± 1.0
*h*_r_ (W/m^2^K)	0.46 ± 0.02	2.62 ± 0.09	5.63 ± 0.17	0.53 ± 0.02	2.95 ± 0.11	6.22 ± 0.18	0.55 ± 0.00	2.98 ± 0.01	6.41 ± 0.03	0.68 ± 0.00	3.52 ± 0.01	7.40 ± 0.07
*h*_r_/*h*_T_ (%)	1.7 ± 0.2	8.9 ± 0.6	15.9 ± 0.2	1.9 ± 0.2	9.4 ± 0.6	16.6 ± 0.2	3.8 ± 0.1	16.7 ± 0.9	32.8 ± 2.3	4.1 ± 0.1	17.4 ± 0.5	32.2 ± 1.7

**Table 10:** Results of ANOVA for total heat transfer coefficient.

Source	*SS*	*df*	*MS*	*F*	*p*-Value	*sig*
Convection	1504.15	1	1504.15	835.96	5.5 × 10^−24^	yes
Surface	326.84	2	163.42	90.82	1.1 × 10^−13^	yes
q_T_	45.11	1	45.11	25.07	2.1 × 10^−5^	yes
Error	55.78	31	1.8			
Total	1931.88	35	55.20			

**Table 11:** Results of ANOVA for convective heat transfer coefficient.

Source	*SS*	*df*	*MS*	*F*	*p*-Value	*sig*
Convection	1620.06	1	1620.06	809.34	0.0000	yes
Surface	13.35	2	6.68	3.34	0.0487	yes
q_T_	28.98	1	28.98	14.48	0.0006	yes
Error	62.05	31	2			
Total	1724.45	35	49.27			

**Table 12:** Results of ANOVA for radiative heat transfer coefficient.

Source	*SS*	*df*	*MS*	*F*	*p*-Value	*sig*
Convection	2.439	1	2.439	34.99	1.6 × 10^−6^	yes
Surface	207.8	2	103.9	1490.46	1.6 × 10^−31^	yes
q_T_	1.756	1	1.756	25.18	2.0 × 10^−5^	yes
Error	2.161	31	0.07			
Total	214.154	35	6.119			
